# Document-Level Biomedical Relation Extraction Using Graph Convolutional Network and Multihead Attention: Algorithm Development and Validation

**DOI:** 10.2196/17638

**Published:** 2020-07-31

**Authors:** Jian Wang, Xiaoyu Chen, Yu Zhang, Yijia Zhang, Jiabin Wen, Hongfei Lin, Zhihao Yang, Xin Wang

**Affiliations:** 1 School of Computer Science and Technology Dalian University of Technology Dalian China; 2 Department of VIP The Second Hospital of Dalian Medical University Dalian China

**Keywords:** biomedical relation extraction, dependency graph, multihead attention, graph convolutional network

## Abstract

**Background:**

Automatically extracting relations between chemicals and diseases plays an important role in biomedical text mining. Chemical-disease relation (CDR) extraction aims at extracting complex semantic relationships between entities in documents, which contain intrasentence and intersentence relations. Most previous methods did not consider dependency syntactic information across the sentences, which are very valuable for the relations extraction task, in particular, for extracting the intersentence relations accurately.

**Objective:**

In this paper, we propose a novel end-to-end neural network based on the graph convolutional network (GCN) and multihead attention, which makes use of the dependency syntactic information across the sentences to improve CDR extraction task.

**Methods:**

To improve the performance of intersentence relation extraction, we constructed a document-level dependency graph to capture the dependency syntactic information across sentences. GCN is applied to capture the feature representation of the document-level dependency graph. The multihead attention mechanism is employed to learn the relatively important context features from different semantic subspaces. To enhance the input representation, the deep context representation is used in our model instead of traditional word embedding.

**Results:**

We evaluate our method on CDR corpus. The experimental results show that our method achieves an F-measure of 63.5%, which is superior to other state-of-the-art methods. In the intrasentence level, our method achieves a precision, recall, and F-measure of 59.1%, 81.5%, and 68.5%, respectively. In the intersentence level, our method achieves a precision, recall, and F-measure of 47.8%, 52.2%, and 49.9%, respectively.

**Conclusions:**

The GCN model can effectively exploit the across sentence dependency information to improve the performance of intersentence CDR extraction. Both the deep context representation and multihead attention are helpful in the CDR extraction task.

## Introduction

Valuable biomedical information and knowledge are still hidden in the exponentially increasing biomedical literature, such as the chemical-disease relation (CDR). Extracting the relation between chemicals and diseases is an important task in biomedical text mining, which plays an important role in various biomedical research studies, such as clinical treatment, drug development, and biomedical knowledge discovery [1-3]. However, extracting CDR from the biomedical literature manually is time-consuming and difficult to keep up-to-date. Thus, the BioCreative V community [4] proposed a task of extracting CDR in the biomedical literature automatically to promote the research on the CDR extraction.

To date, many methods have been proposed for automatic relation extraction between chemicals and diseases, which can be divided into 3 categories: rule-based methods [5], feature-based methods [6-9], and deep neural network-based methods [10-13]. Rule-based methods aim to formulate the heuristic rules for CDR extraction. Lowe et al [5] developed a pattern-based system with some heuristic rules to extract chemical-induced disease (CID) relations within the same sentence. The heuristic rules are used to extract the most likely CID relations when no patterns match a document. Generally, rule-based methods are simple and effective. However, these methods are difficult for application in a new task or dataset. Feature-based methods aim at designing rich features, including semantic and syntactic information. Xu et al [6] utilized text features, including context information and entity information, incorporated with domain knowledge to extract CID relations. Since the syntactic information carried in the dependency graph of the sentence is crucial to CDR extraction, some studies also developed syntactic features. Gu et al [7] utilized various linguistic features to extract CID relations with the maximum entropy model. They leveraged lexical features for both intrasentence and intersentence level relation extraction and developed the dependency features only for intrasentence level relation extraction. Zhou et al [8] utilized the shortest dependency path between chemical and disease entities to extract structured syntactic features. Feature-based methods achieve better performance than rule-based methods. However, traditional feature-based methods only use the dependency trees to extract local syntactic dependencies for the intrasentence level relation extraction, without considering the syntactic dependencies across sentences for the document-level relation extraction. Besides, designing rich features is a time-consuming and laborious task.

In recent years, the deep neural network has been widely used in various natural language processing (NLP) tasks. Some studies have developed deep neural network-based methods for biomedical relation extraction. Long short-term memory (LSTM) models and convolutional neural network (CNN) models are the 2 major neural networks. Zhou et al [10] applied LSTM and CNN models based on traditional word embedding to capture context features for CDR extraction and achieve a good performance. Gu et al [11] proposed a CNN-based model to capture context and dependency features for intrasentence level relation extraction. Nguyen and Verspoor [13] investigated character-based word embedding into the CNN-based relation extraction model. Traditional word embedding such as word2vec cannot vary according to linguistic contexts effectively. Peters et al [14] proposed deep contextualized word representations called ELMo based on a deep bidirectional language model. ELMo can generate a more comprehensive representation for each word based on the sentence context. Therefore, integrating ELMo with a deep neural network may improve the performance of CDR extraction.

In both CNN-based and LSTM-based models, it is hard to distinguish the relevant and irrelevant context features for the relation extraction. A recent study [15] suggested that attention mechanism can capture the most important semantic information for the relation extraction. Vaswani et al [16] introduced a multihead attention mechanism that applied the self-attention mechanism multiple times to capture the relatively important features from different representation subspaces. Thus, multihead attention mechanism can be used to improve the performance of the CDR extraction.

Dependency trees are often used to extract local dependencies for intrasentence level CDR extraction. However, existing studies ignored the nonlocal dependency across sentences, which is crucial for intersentence level CDR extraction. Quirk et al [17] introduced a document graph that can derive features within and across sentences. Thus, we also constructed a document-level dependency graph that can extract dependencies for intrasentence and intersentence level CDR extraction simultaneously. Recently, the graph convolution network (GCN) [18] has been effectively used for encoding document graph information. Thus, GCN can operate directly on the document-level dependency graph to capture long-range syntactic information, which is useful for CDR extraction.

In this study, we evaluated the effectiveness of the deep contextualized word representations, multihead attention mechanism, and GCN in the CDR extraction task. To improve the performance of the intersentence relation extraction, we constructed the document-level dependency graph to capture the dependency syntactic information across sentences. Based on the document-level dependency graph, we proposed a novel end-to-end model to extract CID relations from the biomedical literature. First, we used ELMo, POS embedding, and position embedding to construct the input representation and employed the multihead attention with bidirectional LSTM (BiLSTM) to capture the relatively important context features. Second, we employed the GCN to capture the long-range dependency features based on the document-level dependency graph. Third, we combined the context features and long-range dependency features as the final feature representation and applied a *Softmax* function to implement relation classification. Finally, we evaluated our model on the CDR corpus.

## Methods

### CDR Extraction

The CDR extraction task is a challenging task, which was proposed by the BioCreative V community. The CDR extraction task aims to extract CDR from the biomedical literature automatically and accurately. It is composed of 2 subtasks: (1) disease named entity recognition and normalization and (2) CID relation extraction.

**Figure 1:**
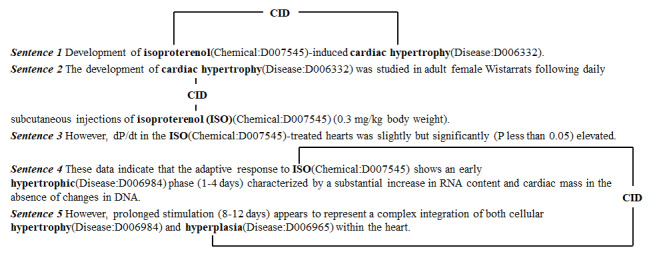
Illustrative examples of CID relation. CID: chemical-induced disease.

In this study, we focused on the CID relation extraction task. The CDR extraction task is a document-level biomedical relation extraction problem, which is different from traditional biomedical relation extraction task. Traditional biomedical relation extraction only considers relation within a single sentence such as protein-protein interaction [19] and drug-drug interaction [20]. However, the CID relation is not only expressed within a single sentence, but it is also expressed across several sentences. Figure 1 shows an illustration of CDR extraction. It is extracted from the CDR corpus whose PMID is 6203632. Among these sentences, the texts in bold mention the chemical and disease entities. In Figure 1, we mark the corresponding entity type and the medical subject headings concept identifiers [21] after the entity mention in the sentence. The chemical D007545 has 2 intrasentence level co-occurrences with disease D006332 in the *sentence 1* and the *sentence 2*, while it has an intersentence level co-occurrence with disease D006965. However, not all occurrences of the chemicals and diseases are considered as a CID relation. For example, the chemical D007545 does not have a CID relation with the disease D006984 in the *sentence 4* because the concept of the disease D006984 is too general to reflect a CID relation.

### Relation Instance Construction

First, we should construct relation instances for both training and testing stages. All the instances generated from the disease and chemical mentions in the document are pooled into 2 groups at the intrasentence and intersentence levels, respectively. The former means that a chemical-disease mention pair is in the same sentence. The latter means that a mention pair is in a different sentence. If the relation between the chemical and disease entity of the mentioned pair is annotated as a CID relation in the document, then this mentioned pair is constructed as a positive instance; otherwise, this mentioned pair is constructed as a negative instance. We applied several effective heuristic rules for both intrasentence and intersentence level instances. The details are as follows.

#### Relation Instance Construction for Intrasentence Level

1. All chemical-disease entity mention pairs that appear in the same sentence are constructed as intrasentence level instances.
2. If multiple mentions refer to the same entity in a sentence, the mentions in the nearest distance should be constructed as an instance.
3. For instance, chemical D007545 and disease D006332 in *sentence 1* form an intrasentence level positive instance, while chemical D007545 and disease D006984 in *sentence 4* form an intrasentence level negative instance.

#### Relation Instance Construction for Intersentence Level

1. Only the chemical-disease entity pairs that are not involved in any intrasentence level are considered as intersentence level instances.
2. If multiple mentions refer to the same entity, the chemical and disease mention in the nearest distance are chosen.

According to our heuristic rules, chemical D007545 in *sentence 4* and disease D006965 in *sentence 5* are regarded as an intersentence level instance because there are no mentions of them in the same sentence. Chemical D007545 in *sentence 1* and disease D006965 in *sentence 5* will be omitted because their distance is not the shortest. Further, chemical D007545 in *sentence 4* and disease D006984 in *sentence 5* are not regarded as an intersentence level instance because chemical D007545 already has intrasentence level co-occurrence with disease D006984 in *sentence 4*.

### Document-Level Dependency Graph

**Figure 2:**
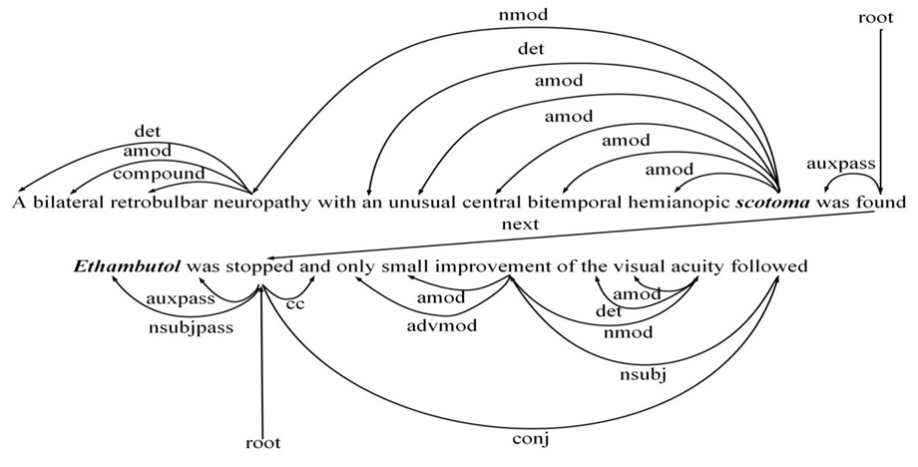
An example of a document-level dependency graph for 2 sentences expressing a CID relation. The chemical and disease entity mention is highlighted in bold. For simplicity, we have omitted self-node edges. CID: chemical-induced disease.

To generate features for entity pairs within and across sentences, we introduce a document-level dependency graph with nodes representing words and edges that show intrasentence and intersentence dependency relations. Figure 2 shows an example of document-level dependency graph for 2 sentences. In this study, we use the following 3 types of intrasentence and intersentence dependency edges.

1. Syntactic dependency edge: The syntactic structure is crucial to biomedical relation extraction. Hence, we use syntactic dependency edges derived from Stanford dependency syntactic parser as intrasentential edges. For instance, “conj” denotes the syntactic relation between the word “stopped” and “followed” in the same sentence.
2. Adjacent sentence edge: Dependencies between sentences are useful for document-level relation extraction. Thus, we consider the sentence as a node in a type of discourse dependency tree. Moreover, we added an edge between the dependency roots of adjacent sentences as an intersentential edge, which is a simple but an effective approach. For instance, “next” denotes the syntactic relation between 2 sentences.
3. Self-node edge: We added self-node edges to all the nodes of the graph in order to enable GCN to not only learn information based on neighbor nodes but also learn the node information itself.

### Model Architecture

**Figure 3:**
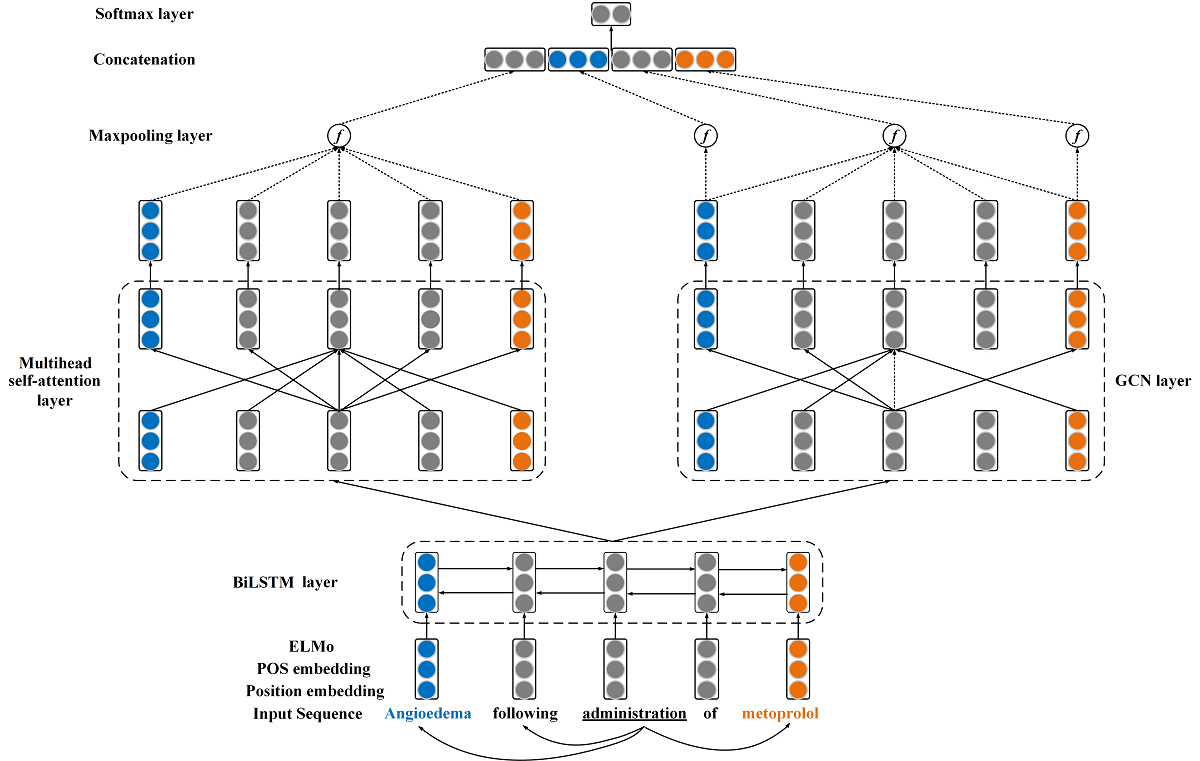
Overview of our model. The input representation consists of ELMo, POS embedding, and position embedding. In the multi-head self-attention layer, we only show the detailed self-attention computation for the word “administration.” In the GCN layer, we only show the detailed graph convolution computation for the word “administration.” BiLSTM: bidirectional long short-term memory; POS: part of speech; GCN: graph convolutional network.

The schematic overview of our model is shown in Figure 3. In short, our model mainly consists of 4 parts: the input representation layer, the BiLSTM layer, the multihead attention layer, and the GCN layer. The inputs of our model are text sequences. The input layer will generate a deep contextualized word representation for each word. Recent studies [22,23] have suggested that the part of speech (POS) and the position of each word are useful for biomedical relation extraction. Hence, we concatenate the deep contextualized word representation and POS and position embedding as the whole word representation. The BiLSTM layer will obtain contextual features from the word representation. The multihead attention layer will apply the self-attention mechanism multiple times to capture the relative semantic features from different representation subspaces. The GCN layer will operate over the document-level dependency graph to capture long-range syntactic features. We employed max pooling over the outputs of the multihead attention layer and the GCN layer and then concatenated these 2 vectors as the final representation. Finally, we employed a fully connected layer and the *Softmax* function to identify the CID relation. Our model will be described in detail in the following section.

#### Input Representation

We used ELMo instead of the traditional word representation in our model. Traditional word representation generates a fixed representation vector for the same word. However, ELMo is the function of the entire input sentence based on a bidirectional language model so that it can generate different representation vectors for the same word according to the different sentence context.

Given that a sequence {*t_1_, t_2_,…..t_N_*} denotes the word tokens in a sentence *S*. Given a token *t_k_*, the forward language model calculates the probability of the token *t_k_* based on the previous tokens {*t_1_*, *t_2,...,_ t_(k-1)_*} of *t_k_* in the sentence *S* as follows:


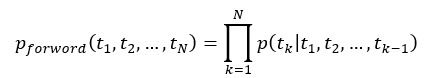
 (1)

Similarly, the backward language model calculates the probability of the token *t_k_* based on the back tokens {*t_1_*, *t_2, …,_ t_(k-1)_*} of *t_k_* in the sentence *S* as follows:


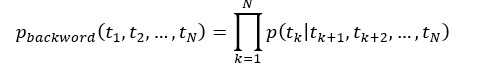
 (2)

Combining the forward and the backward language models as a bidirectional language model, the log-likelihood can be maximized as follows:


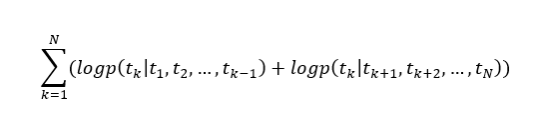
(3)

ELMo can represent the semantic and syntactic information of the word. In our model, we use a linear combination of the hidden state in each layer of the bidirectional language model to generate a deep contextualized representation for words. The POS and the position information of a word are crucial to biomedical relation extraction. Therefore, we also utilize POS embedding and position embedding to enhance the representation ability of the input. The POS embedding represents the POS feature of a word, and the position embedding reflects the relative distance between the word and the target entity. Given a word at position *i,* we obtain its POS embedding *w_p,i_* and position embedding *w_d,i_* based on mapping matrixes *M_p_* and *M_d_*, respectively. Finally, the whole word representations concatenate deep contextualized word representations, POS embedding, and position embedding as follows:

*wi=*[*we,i; wp,i; wd,i*] (4)

#### BiLSTM

The LSTM model is a variant of recurrent neural network models that has been used in many NLP tasks successfully. The LSTM model overcomes the vanishing gradient problem by introducing a gating mechanism [24]. Therefore, it is suitable to capture the long-term dependency feature. The LSTM unit consists of 3 components: the input gate *i_t_*, the forget gate *f_t_*, and the output gate *o_t_*. At the time step *t*, the LSTM unit utilizes the input word *x_t_*, the previous hidden state *h_(t-1)_*, and the previous cell state *c_(t-1)_* to calculate the current hidden state *h_t_* and cell state *c_t_*. The equations are as follows:

*ft=σ(Wfxt+Ufh(t-1)+bf)* (5)

*ot=σ(Woxt+Uoh(t-1)+bo)* (6)

*gt=tanh(Wgxt+Ugh(t-1)+bg)* (7)

*it=σ(Wixt+Uih(t-1)+bi)* (8)

*c_t_*=*f_t_*⊙*c_(t-1)_*+ *i_t_*⊙*g_t_* (9)

*h_t_*= *o_t_*⊙*tanh*(*c_t_*) (10)

where *W, U, b* are the weight and bias parameters, and ⊙ denotes element-wise multiplication. In this study, we use the BiLSTM model that can capture the forward and backward context features simultaneously. The BiLSTM model combines a forward LSTM and a backward LSTM. Given the hidden state of the forward LSTM 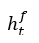 and the hidden state of the backward LSTM 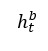, the final hidden state is concatenated as: 
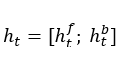


#### Multihead Attention

The BiLSTM model learns the context features from the input sequences automatically and effectively. However, these features make different contributions to the biomedical relation extraction. In our model, we capture the relatively important features by introducing multihead attention mechanism. The essence of multihead attention is applying self-attention mechanism multiple times so that it may let the model learn the relatively important features from different representation subspaces. The self-attention mechanism generates the output based on a query and a set of key-value pairs. The output is the weighted sum of the values, where the weight assigned to each value is computed by applying attention function to the query with the corresponding key. In our study, we deal with the output of the BiLSTM model by multihead self-attention. Further, we use the dot-product attention function instead of the standard additive attention function [25] as follows:


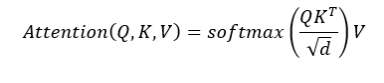
 (11),

where *Q, K, V∈R^n^* represent query, key, and value matrixes, respectively. *d* is the dimension of the output of the BiLSTM model.

The main idea of the multihead attention is applying the self-attention mechanism multiple times. If the multihead attention contains h heads, the *i*-th attention head can be calculated as *head_i_=Attention* (*Q_i_,K_i_,V_i_*). Thus, the final multihead attention is the concatenation of {*head_1,_head_2,_...,head_h_*} as *MultiHead* (*Q,K,V*)*=Concat* (*head_1,_head_2,_...,head_h_*) *W^o^*. The output of the multihead attention layer is a matrix of *R^nat^*.

#### GCN

GCN is an adaptation of CNN [26], which operates on graphs. Given a graph with n nodes, the graph structure can be represented as an adjacency matrix *A*. In this study, we converted the document-level dependency graph into its corresponding adjacency matrix *A*, where *A_ij_=*1 if there is a dependency edge going from token *i* to token *j*; otherwise *A_ij_=*0. The dependency graph can be calculated as an undirected graph [27], which means *A_ij_=A_ji_*. Further, we add a self-node edge to all the nodes in the graph, which means *A_ii_*=1. Since the degree of a node in the dependency graph varies a lot, this may bias the output representation toward favoring high-degree nodes, regardless of the information carried in the node. To solve this issue, we normalize the activations in the graph convolution before feeding it through the nonlinearity. Finally, the graph convolution operation for node *i* at the *l*-th layer where 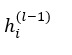 and 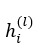 denote the input representation and the output representation of node can be defined as follows:


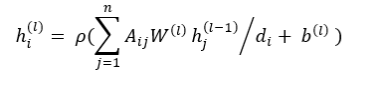
 (12),

where *W^(l)^* is the weight matrix, *b^(l)^* is the bias vector, 
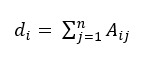
 is the degree of node *i* in the dependency graph, and *ρ* is an activation function (eg, a rectified linear unit).

The GCN model takes the output of the BiLSTM model as the input word representation: 
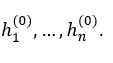
 Then, we stack the graph convolution operation over layers and obtain 
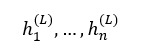
 as the output word representations of the GCN model. Note that the GCN model presented above uses the same parameters for all edges in the dependency graph.

#### Relation Classification

To make use of the output word representation of the GCN model for relation extraction, we generate the sentence representation as follows:

*h_sent_=f* (*h*^(^*^L^*^)^)*=f* (*GCN*(*h*^(^*^0^*^)^) (13)

where *h*^(^*^L^*^)^ denotes the output representations at the last layer *L* of the GCN model, and *f:R^n:R^→R^d^* is a max-pooling function that maps *n* output vectors to the sentence vector.

Inspired by recent studies [28,29], entity information is central to relation classification. Therefore, we also obtain the chemical entity representation *h_c_* as shown in 
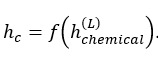
. Similarly, we can obtain the disease entity representation *h_d_*. The feature representation of the whole GCN model is *h_GCN_*=[*h_sent_*; *h_c_*; *h_d_*].

We also obtain the feature representation *h_att_* from the output of the multihead attention layer by applying max pooling to the multihead attention matrix. We concatenate *h_GCN_* and *h_att_* to form the final representation *h_final_*=[*h_GCN_*; *h_att_*] for relation classification. Then, the final representation is fed into a 2-layer perceptron as follows:


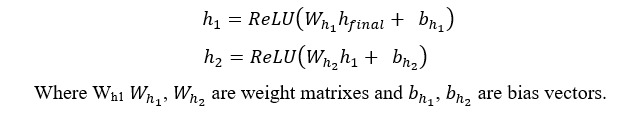
 (14) and (15).

Finally, the hidden representation *h_2_* is fed to a *Softmax* function to calculate the confidence of the CID relation:

*o=softmax* (*W_o_h_2_+b_o_*) (16)

where *o* is the output, *W_o_* is the weight matrix, and *b_o_* is the bias vector.

## Results

### Dataset

We evaluated our model on the CDR corpus, which was released by the BioCreative V task. The CDR dataset is the benchmark dataset for the CID relation extraction task, which consists of 1500 PubMed abstracts—500 each for training, development, and test set. Table 1 shows the details of the dataset.

**Table 1:** Statistics of the chemical-disease relation dataset.

Task dataset	Abstracts (n=1500)	Chemical-induced disease relations (n=3116)
Training	500	1038
Development	500	1012
Test	500	1066

In this study, the gold entity annotations provided by BioCreative V were used to evaluate our model. All the comparison methods reported in this paper were evaluated with gold entity annotations. Therefore, it is fair and comparable. Further, we measured the CID relation extraction performance with precision, recall, and F-measure.

### Experimental Settings

The dimensions of POS embedding and position embedding are both 100. The dimension of ELMo is 1024. The dimensions of the LSTM hidden layer and the GCN layer are 500 with the dropout proportion of 0.5. The dimensions of 2-layer perceptron are also 500 with the dropout proportion of 0.5. Our model was trained by Adam [30] with a learning rate of 0.001 and a minibatch size of 32. In addition, our model was implemented based on an open-source deep learning library PyTorch [31]. We used StanfordNLP [32] to obtain the POS of the word and the dependency tree. Further, we used the pretrained ELMo representations for the deep contextualized word representations.

### Experimental Results

#### Effect of Input Representation

We evaluated the effectiveness of the input representation of our model. We used the same model that we proposed and changed the input representations. The comparison performance of the different input representations is presented in Table 2.

**Table 2:** The effect of the input representation on performance.

Input representation	Precision (%)	Recall (%)	F-measure (%)
Word^a^	47.3	71.7	57.0
Word+position^b^	49.1	71.4	58.2
Word+position+POS^c^	51.6	71.8	*60.1*
ELMo^d^	57.0	67.4	61.8
ELMo+position^e^	54.2	74.9	62.9
ELMo+position+POS^f^	56.3	72.7	*63.5*
BioBERT+position+POS^g^	57.9	70.1	63.4

^a^The input representation of the model is the word embedding, which is pretrained by word2vec.

^b^The input representation of the model is the concatenation of the word embedding and position embedding.

^c^The input representation of the model is the concatenation of the word embedding, position embedding, and part of speech (POS) embedding. The F-measure (%) for this representation was an important finding.

^d^The input representation of the model is the deep contextualized word representation.

^e^The input representation of the model is the deep contextualized word representation and position embedding.

^f^The input representation of the model is the deep contextualized word representation, position embedding, and POS embedding. The F-measure (%) for this representation was an important finding.

^g^The word representation is generated from the last hidden layer of the bidirectional encoder representations from transformers for biomedical text mining (BioBERT) [33] in a feature-based approach, which means that the parameters of the BioBERT are not fine-tuned. The input representation of the model is the BioBERT word representation, position embedding, and POS embedding.

In Table 2, we can observe that the model achieves an F-measure of 57.0% when we only use the pretrained word embedding as the input representation. When we concatenate the pretrained word embedding and position embedding, the F-measure is improved from 57.0% to 58.2%, which yields a 1.2% improvement. When we concatenate the pretrained word embedding, position embedding, and POS embedding as the input representations, we yield another 1.9% improvement compared with only using the pretrained word embedding and position embedding. The result indicates that both POS and position features are effective for the CID relation extraction. The deep contextualized word representation ELMo significantly outperforms the pretrained word embedding and yields a 4.8% improvement in the F-measure. The result indicates that ELMo can generate a more comprehensive representation for the word according to the sentence context, which results in a better CDR performance. Similarly, combining the position and POS embedding with the deep contextualized word representation can further improve the performance. When we concatenate the deep contextualized word representation, position embedding, and POS embedding as the input representation, we achieve the best F-measure of 63.5%. We also use the word representations generated from the bidirectional encoder representations from transformers for biomedical text mining in a feature-based approach and achieve an F-measure of 63.4%, which is similar to using ELMo.

#### Effect of the Attention Mechanism

We evaluated the effectiveness of the multihead self-attention mechanism. We used the same model architecture that we proposed, but we dealt with the output of BiLSTM by different attention mechanisms. The attention mechanism is divided into 2 categories: single-head attention mechanism and multihead attention mechanism. In single-head attention mechanism, we use 3 types of attention function: additive attention, general attention, and scaled dot-product attention, as shown below.


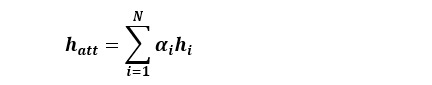
 (17)


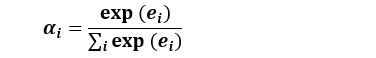
 (18)


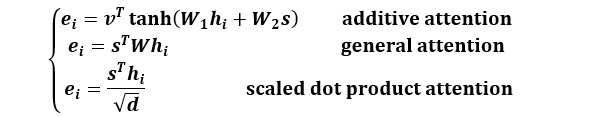
 (19)

where *h_i_* is the output of the BiLSTM, *W_1_, W_2_, s, v* are the parameter matrixes, and *d* is the dimension of the output of the BiLSTM model. The formula of the multihead attention is described in formula (11). The comparison performance of the different attention mechanism is presented in Table 3.

**Table 3:** The effect of the attention mechanism on performance.

Attention mechanism	Precision (%)	Recall (%)	F-measure (%)
Without attention	55.1	71.3	62.2
Additive attention	55.9	70.3	62.3
General attention	55.3	71.8	62.5
Scaled dot-product attention	54.9	73.3	62.8
Multihead attention	56.3	72.7	63.5

In Table 3, we can see that using the attention mechanism can improve the performance of the CID relation extraction. The multihead attention mechanism is more helpful than other single-head attention mechanisms. This suggests that the multihead attention mechanism can capture more valuable features from different representation subspaces.

#### Effect of the Attention Heads

We evaluated the effectiveness of the number of heads of the multihead attention mechanism. In this comparative experiment, we used the deep contextualized word representation, position embedding, and POS embedding as the input representation, and the dimensions of query, key, and value are the same. As shown in Table 4, we only varied the number of heads of the multihead attention.

**Table 4:** The effect of the attention heads on performance.

Heads (n)	Precision (%)	Recall (%)	F-measure (%)
2	57.2	68.2	62.2
4	56.9	70.6	63.0
5	56.3	72.7	63.5
8	57.0	70.2	62.9
10	54.4	75.4	63.2

In Table 4, we can see that the multihead attention mechanism can effectively improve the performance of the CID relation extraction. We can observe that the F-measure ranges from 62.2% to 63.5% when setting a different number of heads. When the number of heads is too little or too large, the performance will drop off. In short, we achieve the best F-measure of 63.5% when we set the number of heads as 5.

### Ablation Study

To examine the contributions of the 2 main components, namely, multihead attention layer and GCN layer, we ran an ablation study. The experimental results are shown in Table 5. The results contain intrasentence level, intersentence level, and relation merging, which means that merging the intrasentence and intersentence level results in the final document-level result.

**Table 5:** An ablation study for our model.a

Model	Intrasentence level			Intersentence level			Relation merging		
	Precision (%)	Recall (%)	F-measure (%)	Precision (%)	Recall (%)	F-measure (%)	Precision (%)	Recall (%)	F-measure (%)
Without multihead attention	58.2	82.9	68.4	44.7	44.3	44.5	55.1	71.3	62.2
Without GCN^b^	62.6	74.1	67.9	43.6	48.4	45.9	57.1	66.4	61.4
Our model	59.1	81.5	*68.5*	47.8	52.2	*49.9*	56.3	72.7	*63.5*

^a^The values in italics indicate significant findings.

^b^GCN: graph convolutional network.

We can observe that removing either the multihead attention layer or the GCN layer reduces the performance of the model. This suggests that both layers can learn effective features. When we remove the multihead attention layer and the GCN layer, the F-measure drops by 1.3% and 2.1%, respectively. In particular, we can observe that adding either the multihead attention layer or the GCN layer improves the performance in the intersentence level relation extraction by a large margin. When we remove the multihead attention layer and the GCN layer, the intersentence level F-measure drops by 5.4% and 4.0%, respectively. This suggests that the multihead attention layer can capture the relatively important features from different representation subspaces and the GCN layer can capture long-range syntactic features for intersentence level relation extraction.

### Comparison with Related Work

We compared our model with several state-of-the-art methods of the CID relation extraction. These methods are divided into 2 categories: methods without additional resources (without knowledge bases) and methods using additional resources (with knowledge bases). These following methods have been summarized in Table 6.

1. Pattern rule-based: Lowe et al [5] developed a pattern-based system with some heuristic rules to extract CID relations within the same sentence, and they achieved an F-measure of 60.8%.
2. Maximum entropy model: Gu et al [7] developed a machine learning-based system that utilized simple but effective manual linguistic features with the maximum entropy model. They built rich manual features for intrasentence level and intersentence level instances. They achieved an F-measure of 58.3%.
3. LSTM+ support vector machine (SVM): Zhou et al [10] developed a hybrid system, which consists of a feature-based model that utilized flat features and structure features with SVM and a neural network model based on LSTM. Their model achieved an F-measure of 56.0%. After using additional postprocessing heuristic rules, they achieved a 5.3% improvement in the F-measure.
4. CNN+maximum entropy: Gu et al [11] proposed a maximum entropy model for intersentence level relation extraction and a CNN model for intrasentence level relation extraction. They achieved an F-measure of 60.2%. They also used additional postprocessing heuristic rules to improve performance that increases the F-measure to 61.3%.
5. Biaffine Relation Attention Network: Verga et al [12] proposed this based on the multihead self-attention model, which can predict relationships between all the mentioned pairs in the document. The model achieved an F-measure of 62.1%.
6. Graph convolutional neural network: Sahu et al [18] proposed a labelled edge graph convolutional neural network model on a document-level graph. The model achieved an F-measure of 58.6%.
7. SVM_Xu: Xu et al [6] explored 4 different knowledge bases to extract the knowledge features and achieved an F-measure of 67.2%.
8. SVM_Pons: Pons et al [9] extracted 3 sets of features, which are prior knowledge and statistical and linguistic information from the document. They achieved an F-measure of 70.2%.
9. Knowledge-guided convolutional network: Zhou et al [34] proposed a CNN that integrated both relation representations and entity representations learned from knowledge bases. The model achieved an F-measure of 71.3%.

**Table 6:** Comparisons with related work.

Category and method			Precision (%)	Recall (%)			F-measure (%)
**Without knowledge bases**							
	**Lowe et al [5]**						
		Pattern rule-based	59.3	62.3			60.8
	**Gu et al [7]**						
		ME^a^	62.0	55.1			58.3
	**Zhou et al [10]**						
		LSTM+SVM^b^	64.9	49.3			56.0
		LSTM+SVM+PP^c^	55.6	68.4			61.3
	**Gu et al [11]**						
		CNN+ME^d^	60.9	59.5			60.2
		CNN+ME+PP	55.7	68.1			61.3
	**Verga et al [12]**						
		BRAN^e^	55.6		70.8	62.1	
	**Sahu et al [18]**						
		GCNN^f^	52.8		66.0	58.6	
	**Our study**						
		GCN^g^+Multihead attention	56.3		72.7	63.5	
**With knowledge bases**							
	**Xu et al [6]**						
		SVM	65.8		68.6	67.2	
	**Pons et al [9]**						
		SVM	73.1		67.6	70.2	
	**Zhou et al [34]**						
		KCN^h^	69.7		72.9	71.3	

^a^ME: maximum entropy model.

^b^LSTM+SVM: long short-term memory+support vector machine.

^c^LSTM+SVM+PP: long short-term memory+support vector machine+postprocessing.

^d^CNN+ME: convolutional neural network+maximum entropy model.

^e^BRAN: biaffine relation attention network.

^f^GCNN: graph convolutional neural network.

^g^GCN: graph convolutional network.

^h^KCN: knowledge-guided convolutional networks.

In Table 6, the deep neural network-based methods achieved competitive performance in the CID relation extraction task. For example, Sahu et al [18] used GCN to capture dependency information and achieved an F-measure of 58.6%. Compared with other deep neural network-based methods, we not only employed the multihead attention to capture the relatively important semantic features but also used the GCN to capture the valuable syntactic features from the document-level dependency graph automatically and effectively. We also observed that some studies [7,10,11] designed and extracted rich semantic and syntactic features for the relation extraction task and used additional postprocessing heuristic rules to improve performance. Our method is an end-to-end neural network-based model and achieves a high F-measure of 63.5% without using postprocessing heuristic rules. As shown in Table 6, the methods with knowledge bases outperform the methods without knowledge bases significantly. This suggests that prior knowledge is much useful for CID relation extraction. In this study, we focus on the effectiveness of GCN and multihead attention mechanism rather than the prior knowledge. We will attempt to integrate the biomedical knowledge to further improve the performance of our method in our future work.

### Visualization of Multihead Attention Mechanisms

**Figure 4:**
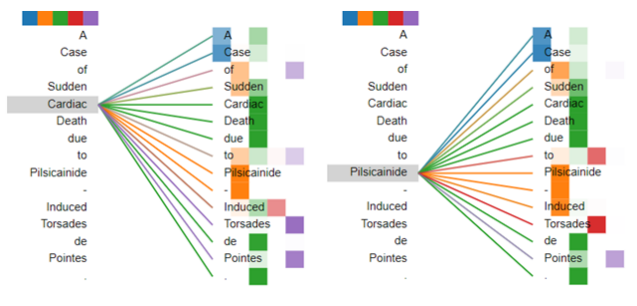
Examples of the multi-head self-attention mechanism. Attentions here shown only for the words "Cardiac" and "Pilsicainide." Different colors represent different heads.

To understand our multihead self-attention mechanism clearly, we visualized the attention weights of an example sequence in Figure 4. Different colors represent different heads. The darker the color is, the higher the attention weight is. In Figure 4, the word pays different levels of attention to different words in different heads. For the word “Cardiac,” the word “Pilsicainide” has the higher weight score in the second head; however, the words “Torsades” and “Pointes” have the higher weight score in the last head. For the word “Pilsicainide,” the words “Cardiac” and “Death” have the higher weight score in the third head; however, the word “Torsades” has the higher weight score in the fourth head. Thus, the multihead self-attention mechanism can make the model capture the relatively important features from different representation subspaces.

### Error Analysis

To understand our model better, we performed an error analysis on the output of our final results. There are the 2 main types of errors: false positive errors and false negative errors. We list some examples to analyze the errors. In false positive errors, some instances are nonrelations but are mistaken as CID relations. For the sentences “Carbamazepine (Chemical: D002220)-induced cardiac dysfunction (Disease: D006331)” and “A patient with sinus bradycardia and atrioventricular block (Disease: D054537) induced by carbamazepine (Chemical: D002220),” the disease D006331 is the hypernym of the disease D054537. According to the labeling rules of the CDR corpus, we need to extract the most specific relations. Thus, the first sentence does not express a CID relation and the second sentence expresses a CID relation. However, our model extracts a CID relation between the chemical D002220 and the disease D006331 in the first sentence incorrectly because the first sentence is the common sentence pattern that expresses a CID relation. In false negative errors, several CID relations are not recognized. One of the main reasons for some intersentence level instances to be removed by the heuristic rules in the relation instance construction stage is because the sentence distance is more than 3. In the future, we will consider preferable preprocessing and postprocessing techniques to solve the above problems.

## Discussion

In this paper, we propose a novel end-to-end neural network based on GCN and multihead attention. The document-level dependency graph is constructed to capture the dependency syntactic information across sentences. We applied GCN to capture the long-range dependency syntactic features, which can improve the performance of intersentence level relation extraction. Further, we employed the multihead attention mechanism to capture the relatively important context features from different semantic subspaces. ELMo is used in our model to enhance the input representation. We evaluate the effectiveness of ELMo, multihead attention mechanism, and GCN on the BioCreative V CDR dataset. Experimental results show that ELMo, multihead attention, and GCN can significantly improve the performance of the CDR extraction. Our method achieves an F-measure of 63.5%, which is superior to other state-of-the-art methods. There are many large-scale knowledge bases such as the Comparative Toxicogenomics Database, Unified Medical Language System, Medical Subject Headings, UniProt, and the commercial system Euretos Knowledge Platform. These knowledge bases contain a large amount of structured data in the form of triples (entity, relation, entity), wherein relation represents the relationship between 2 entities. Some studies suggest that integrating the structured information from the knowledge bases may improve the performance of the CDR extraction. In future studies, we will integrate the biomedical knowledge to further improve the performance of our method.

## Abbreviations

BiLSTM: bidirectional long short-term memory
CDR: chemical-disease relation
CID: chemical-induced disease
CNN: convolutional neural network
GCN: graph convolutional network
LSTM: long short-term memory
NLP: natural language processing
POS: part of speech
SVM: support vector machine
